# Supplementary material for: Transmission of Escherichia coli from Manure to Root Zones of Field-Grown Lettuce and Leek Plants
Source: Microorganisms. 2021 Nov 3;9(11):2289. doi: 10.3390/microorganisms9112289 (PMC8622635; doi:10.3390/microorganisms9112289)
Supplement: Supplementary file 1 [file microorganisms-09-02289-s001.zip › microorganisms-1401014-SI.pdf]

Table S1. Primers, probes and gBlock used in TaqMan analysis of DNA extracts derived from manure, and bulk and rhizosphere soils.

Oligonucleotide/ Gblock *	Sequence 5’ to 3’	Dye
*bla*CTX-M-55-Fw	GTACCGAGCCGACGTTAAA	-
*bla*CTX-M-55-Rv	TTTACCCAGCGTCAGATTCC	-
*bla*CTX-M-55-P	ATACCACTTCACCTCGGGCAATGG	FAM
*qnr*S1-Fw	TATCGAAGGCTGCCACTTTG	-
*qnr*S1-Rv	CACACGCACGGAACTCTATAC	-
*qnr*S1-P	TCCAACAATGCCAACTTGCGATGG	FAM
*rec*A-Fw	GGATGTGGAAACCATCTCTACC	-
*rec*A-Rv	GTCCGTAGATTTCGACGATACG	-
*rec*A-P	TCGCTTTCACTGGATATCGCGCTT	HEX
gBlock	CCATGGATGTGGAAACCATCTCTACCGGTTCGCTTTCACTGGATATCGCGCTTGGGGCAGGTGGTCTGCCGATGGGCCGTATCGTCGAAATCTACGGACCGGAGTGATATCGAAGGCTGCCACTTTGATGTCGCAGATCTTCGTGATGCAAGTTTCCAACAATGCCAACTTGCGATGGCAAACTTCAGTAATGCCAATTGCTACGGTATAGAGTTCCGTGCGTGTGATTTGACCGTACCGAGCCGACGTTAAACACCGCCATTCCGGGCGATCCGCGTGATACCACTTCACCTCGGGCAATGGCGCAAACTCTGCGGAATCTGACGCTGGGTAAAGCAT	

* Fw, forward primer; Rv, reverse primer; P, probe; FAM (5(6)-carboxyfluorescein), HEX (Hexachlorofluorescein), fluorescent dyes; DNA stretches in gBlock sequence marked in red are targeted by the *rec*A Taqman system, in blue by that of *qnr*S1 and in green by the one of *bla*CTX-M-55. Zen and Iowa Black (IDT, Coralville, Iowa) were used as double quenchers in TaqMan assays.

GBlock:

CCATGGATGTGGAAACCATCTCTACCGGTTCGCTTTCACTGGATATCGCGCTTGGGG
CAGGTGGTCTGCCGATGGGCCGTATCGTCGAAATCTACGGACCGGAGTGATATCGAA
GGCTGCCACTTTGATGTCGCAGATCTTCGTGATGCAAGTTTCCAACAATGCCAACTTG
CGATGGCAAACTTCAGTAATGCCAATTGCTACGGTATAGAGTTCCGTGCGTGTGATT
TGACCGTACCGAGCCGACGTTAAACACCGCCATTCCGGGCGATCCGCGTGATACCAC
TTCACCTCGGGCAATGGCGCAAACTCTGCGGAATCTGACGCTGGGTAAAGCAT

DNA stretches marked in red are targeted by the recA Taqman system, in blue by that of qnrS1 and in green by the one of blaCTX-M-55.
