# Supplementary material for: Transmission of Escherichia coli from Manure to Root Zones of Field-Grown Lettuce and Leek Plants
Source: Microorganisms. 2021 Nov 3;9(11):2289. doi: 10.3390/microorganisms9112289 (PMC8622635; doi:10.3390/microorganisms9112289)
Supplement: Supplementary file 1 [file microorganisms-09-02289-s001.zip › Supplementary figures.pptx]

## Slide 1
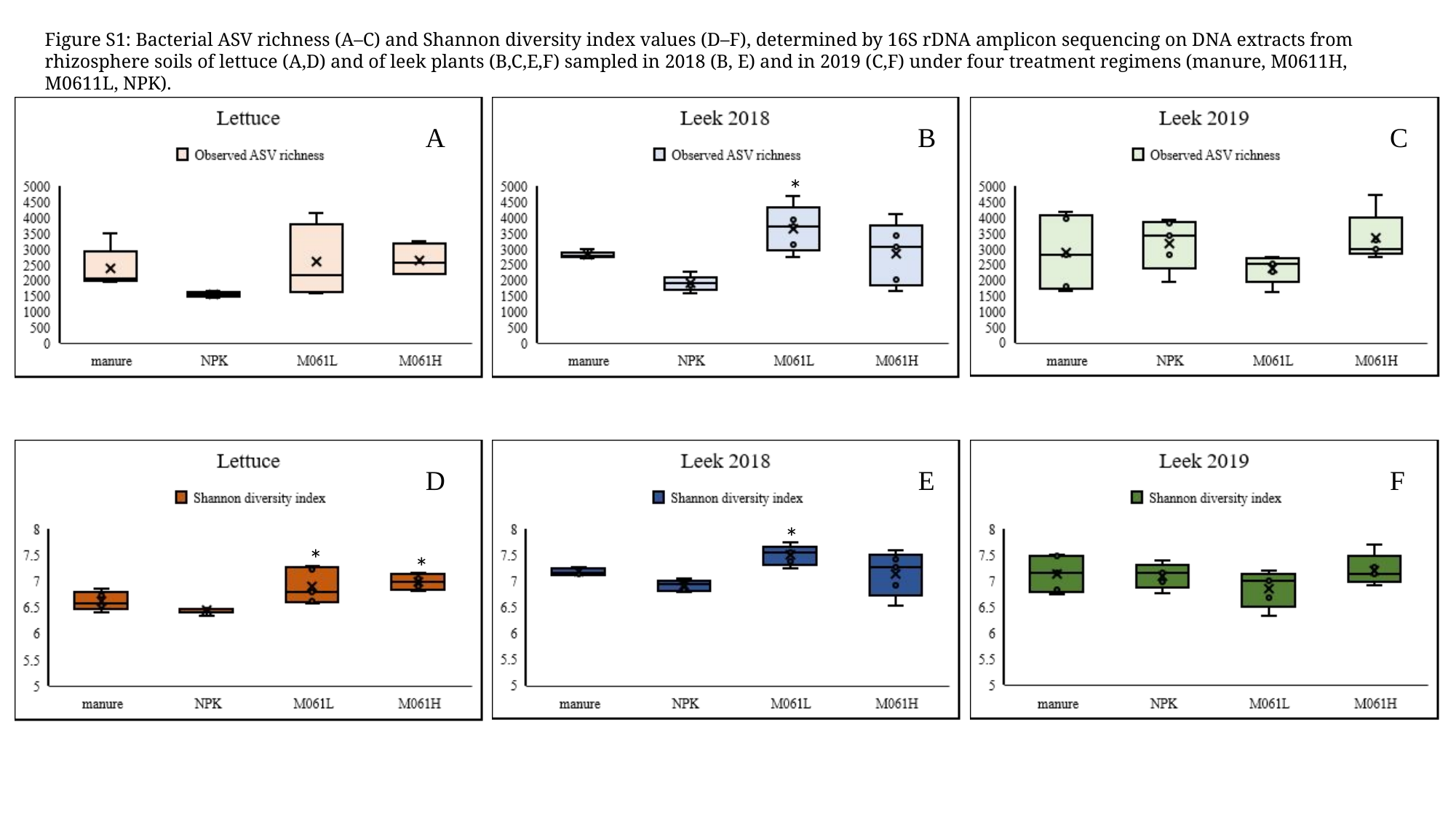

Figure S1: Bacterial ASV richness (A–C) and Shannon diversity index values (D–F), determined by 16S rDNA amplicon sequencing on DNA extracts from rhizosphere soils of lettuce (A,D) and of leek plants (B,C,E,F) sampled in 2018 (B, E) and in 2019 (C,F) under four treatment regimens (manure, M0611H, M0611L, NPK).
A
B
C
*
D
E
F
*
*
*

## Slide 2
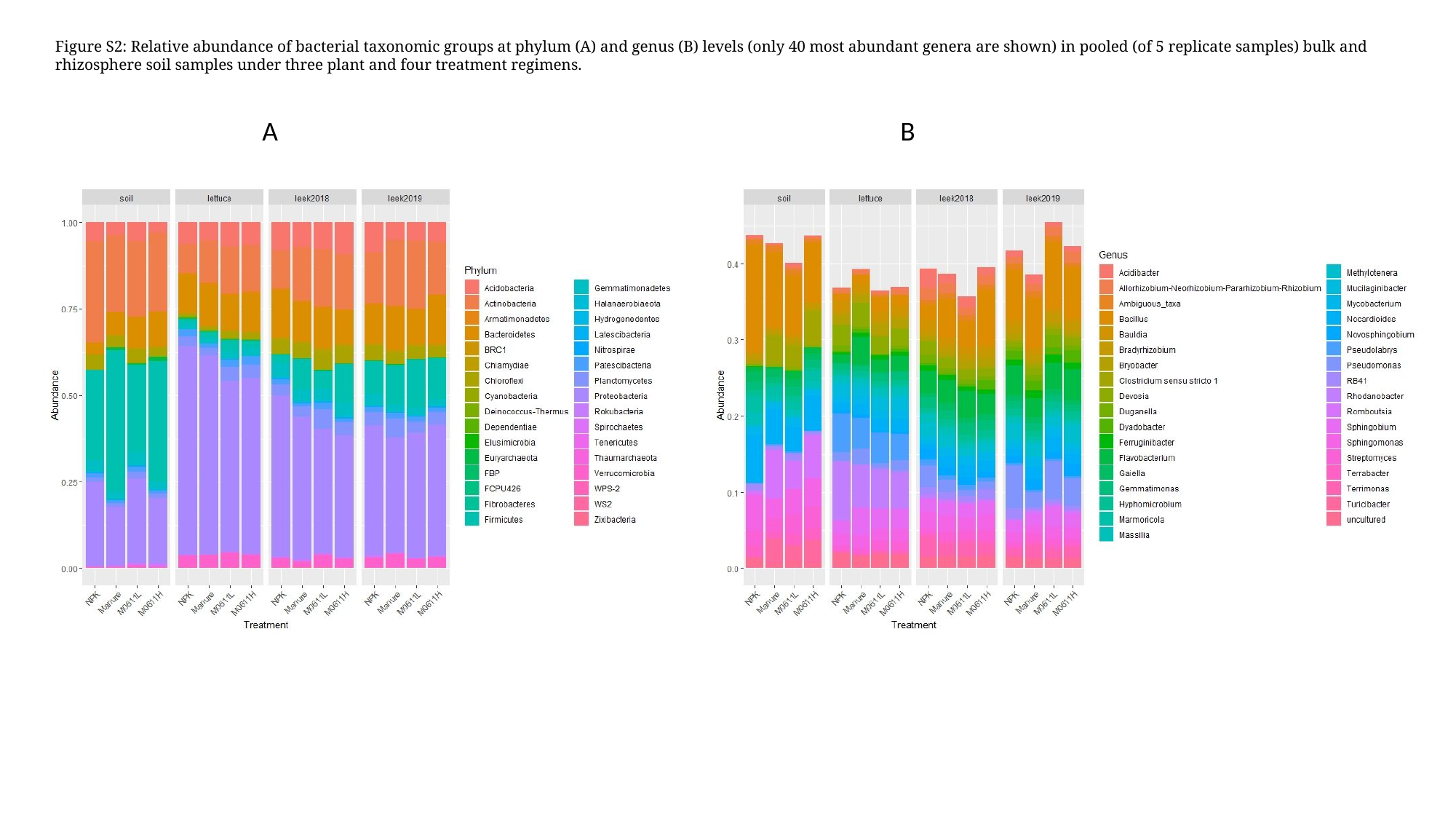

Figure S2: Relative abundance of bacterial taxonomic groups at phylum (A) and genus (B) levels (only 40 most abundant genera are shown) in pooled (of 5 replicate samples) bulk and rhizosphere soil samples under three plant and four treatment regimens.
A
B

## Slide 3
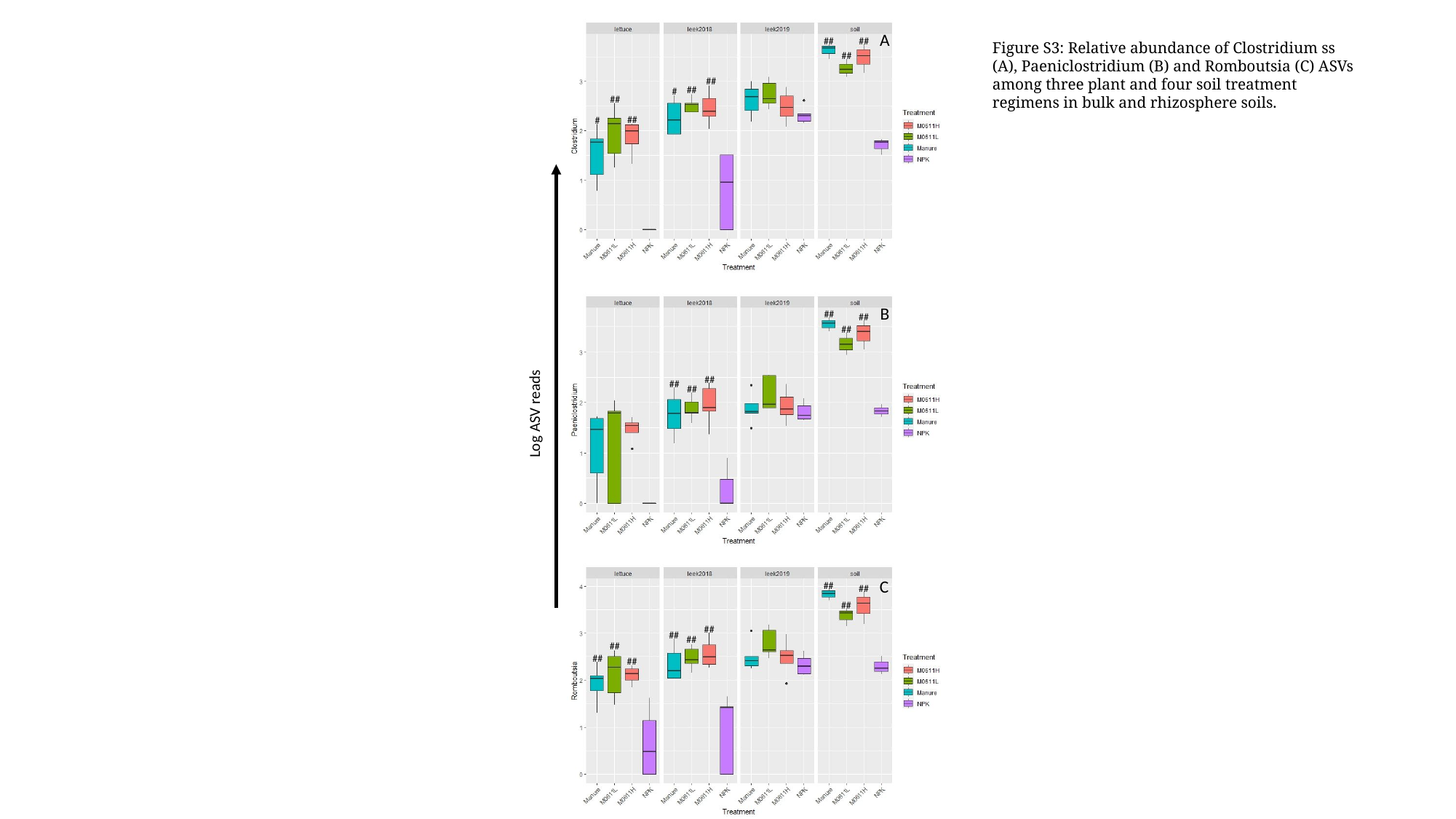

A
##
##
##
##
##
#
##
##
#
B
##
##
##
##
##
##
C
##
##
##
##
##
##
##
##
##
Figure S3: Relative abundance of Clostridium ss (A), Paeniclostridium (B) and Romboutsia (C) ASVs among three plant and four soil treatment regimens in bulk and rhizosphere soils.
Log ASV reads
